# Craniofacial anthropometric investigation of relationships between the nose and nasal aperture using 3D computed tomography of Korean subjects

**DOI:** 10.1038/s41598-020-73127-8

**Published:** 2020-09-30

**Authors:** Joon Yeol Ryu, Ki-Su Park, Min-Ji Kim, Ji-Su Yun, U-Young Lee, Sang-Seob Lee, Byung-Yoon Roh, Jeong-Uk Seo, Chang-Un Choi, Won-Joon Lee

**Affiliations:** 1 Division of Forensic Medicine, National Forensic Service, Wonju, 26460 Republic of Korea; 2 Department of Anatomy, College of Medicine, Catholic Institute for Applied Anatomy, Catholic University of Korea, Seoul, 06591 Republic of Korea; 3 Department of Forensic Medicine, National Forensic Service Seoul Institute, Seoul, 08063 Republic of Korea

**Keywords:** Biological anthropology, Bone development

## Abstract

This study investigated the relationships of morphology and locations of the nose and nasal aperture by using major craniofacial landmarks on the human skull and face for craniofacial reconstruction/approximation of Koreans. In the frontal view, the positions of bony landmarks on the skull, including the nasal aperture, were correlated with the positions of nasal landmarks vertical to the transverse plane. In profile, the positions of bony landmarks on the skull were correlated with the positions of nasal landmarks horizontal to the coronal plane. Overall, 26 of the 76 measurements demonstrated significant correlations between the corresponding landmarks on the nose and nasal aperture. Simple regression equations were produced from the results. This study showed that the nose and nasal aperture are significantly related to each other in terms of their morphology and location in Koreans. The prediction guidelines, produced as regression formulas, can be applied to craniofacial reconstruction/approximation and bio-anthropological research of Korean skulls. The study results can also be used clinically in rhinoplasty and nasal reconstruction surgery.

## Introduction

Craniofacial reconstruction/approximation is a face recreation tool that is used in forensic investigations to identify unknown skulls^1^. Reconstruction of the nose is a crucial step in craniofacial reconstruction because the eyes fixate on the area around the centre of the nose for recognition^2^. Many studies have examined the use of facial parts such as the mouth and eyes for craniofacial reconstruction^3–5^, including predictions of nose morphology using a variety of methods^6–12^.

In a study of published methods, Stephan et al. found that the methods of George^10^ and Prokopec and Ubelaker^11^ performed well, while those of Gerasimov^7^ and Krogman^8^ performed poorly^5^. Rynn et al. found that the two-tangent method of Gerasimov^7^ was optimal for prediction of the nasal tip, whereas others methods performed poorly in terms of nasal profile construction, or were of questionable accuracy^13^. While the methods of Stephan et al. and Rynn et al. are generally accurate, their accuracies have varied among sample groups^14^.

In addition to the accuracy of nose prediction, the overall rotation of facial features, including the anterior view in nose reconstruction, affects the recognition of reconstructed unidentified skulls. However, most published nose-prediction guidelines emphasise the projection of the nose in profile, especially the tip of the nose; other parts of the nose, such as the nasal root and ala nasi, are considered less important.

Because the ethnicities of the subjects in most studies are heterogeneous and few Koreans have been included, the application of these methods to the craniofacial reconstruction of Koreans has involved uncertainty. Rynn’s method has been tested in different ethnic populations and has demonstrated considerable variation, suggesting differences in outcomes among ethnicities^15,16^. Thus, when developing nose-morphology prediction methods, the method should examine samples from a single ethnic group. Therefore, the present study examined data from the Korean population, with the aim of predicting the noses of Koreans. Studies of nose morphology in the Korean population have revealed the characteristics of the Korean nose^17–22^. Moreover, Lee et al. discussed the advantages of three-dimensional computed tomography (3D CT) for predicting the position of the nose; they developed multiple regression equations for Koreans^22^.

**Figure 1:**
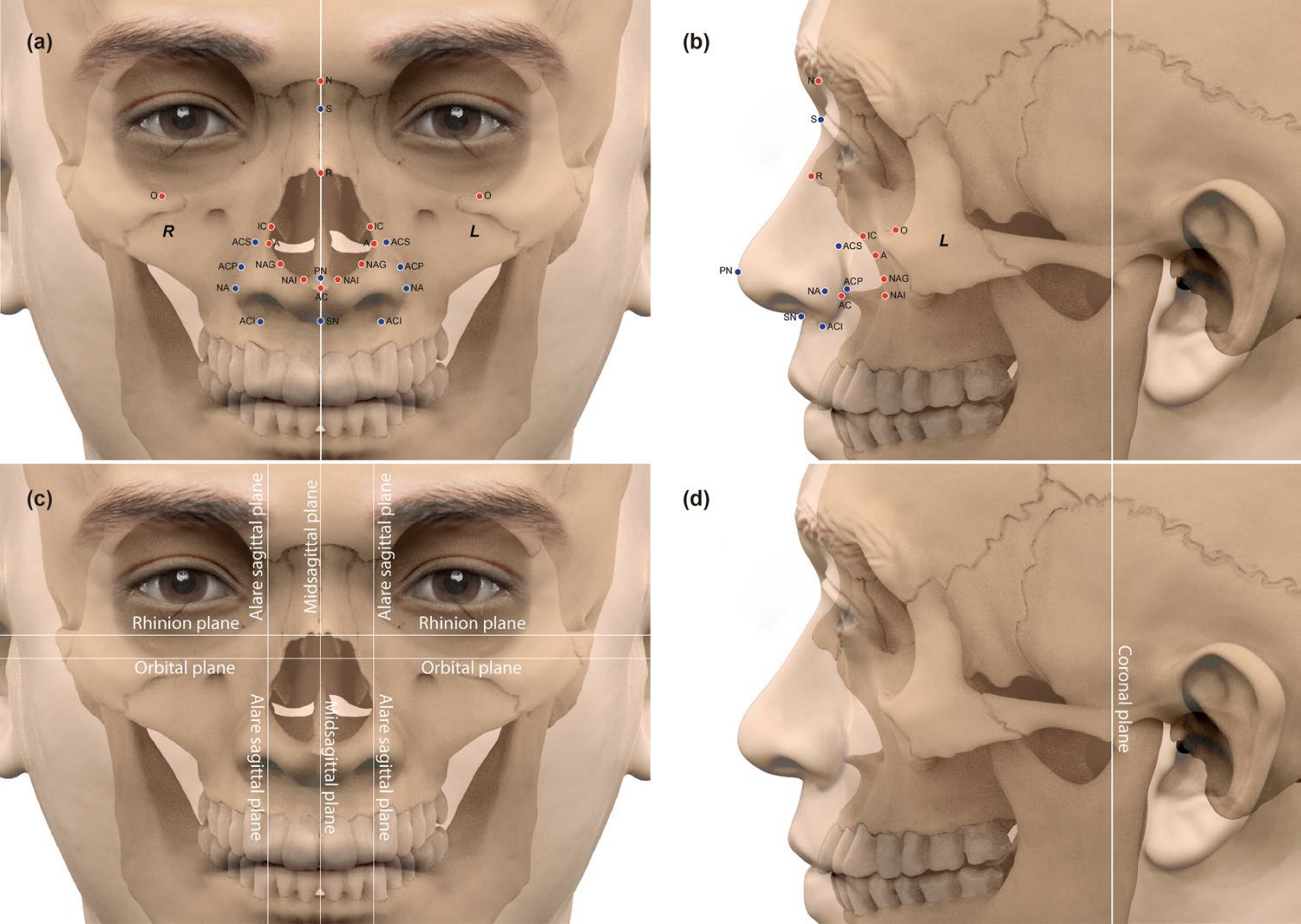
Positions of the craniofacial landmarks and reference planes. Craniofacial landmarks in the (**a**) anterior and (**b**) profile views; reference planes in the (**c**) anterior and (**d**) profile views. Red dots, bony landmarks; blue dots, facial soft tissue landmarks. The face and skull images used in this manuscript were modified from CT images of the author WJL.

**Figure 2:**
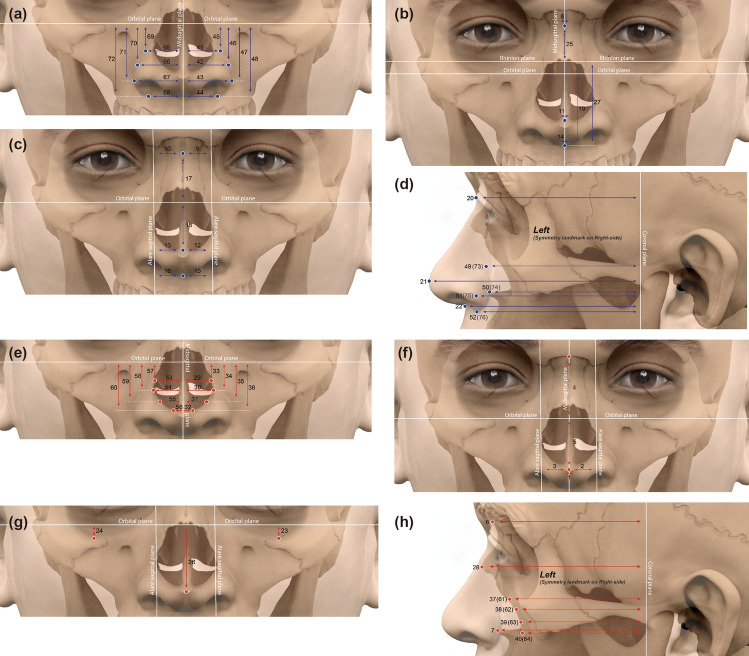
Views showing the 76 measurement sections. Facial measurement sections in the (**a**–**c**) anterior and (**d**) profile views; bony measurement sections in the (**e**–**g**) anterior and (**h**) profile views. Red dots, bony landmarks; blue dots, facial soft tissue landmarks.

In this study, 3D CT images of Koreans were used to examine the relationship between the nose and nasal aperture to develop a more accurate nose-prediction method. To measure distances, landmarks were arrayed along the nose and nasal piriform aperture, while reference planes were used to subdivide the skulls and faces of the subjects into sections for analysis (Figs. 1 and 2).

## Results

### Data analysis and regression equations

**Figure 3:**
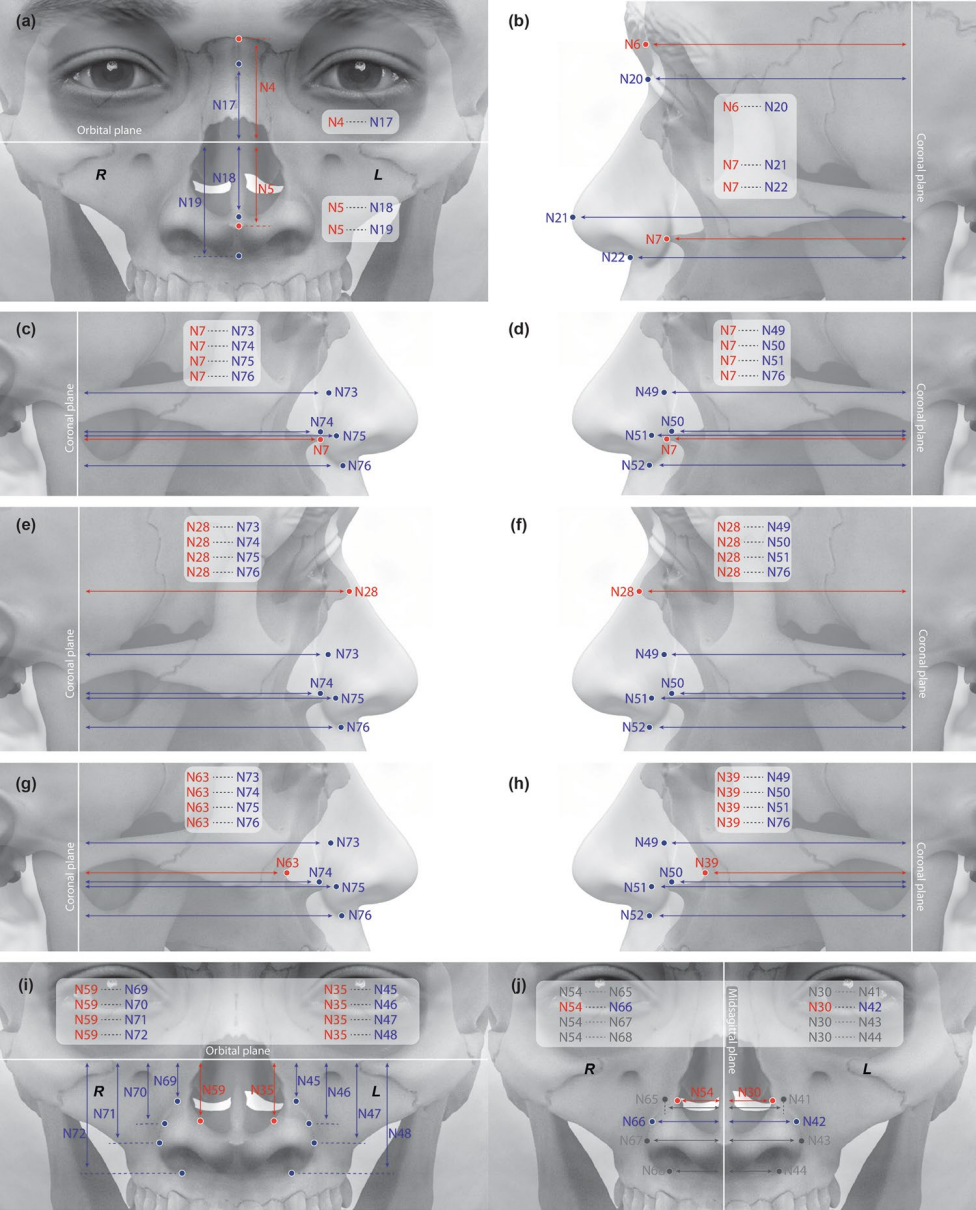
The 40 pairs of measurements with positive correlations at the 0.05 level. Each bony tissue (red) location is positively correlated with a facial soft tissue landmark (blue). Positive correlations in the (**a**) anterior view for nasal height, (**b**) lateral view for nasal depth, (**c**–**h**) lateral view for ala nasi depth, (**i**) anterior view for ala nasi height, and (**j**) anterior view for nasal width.

For the 76 measurements, Pearson’s correlation analysis revealed 40 positive correlations at the 0.05 significance level between bony and facial soft tissue pairs (Fig. 3, Supplementary Table A): three pairs of bony and soft tissue measurements in the anterior view for nasal height; three pairs of median bony and soft tissue measurements and 12 pairs each (total 24 pairs) of bilateral bony and soft tissue measurements in the left and right lateral views for nasal depth; eight pairs of bony and soft tissue measurements in the anterior view for the left and right ala nasi heights; and two pairs of bony and soft tissue measurements in the anterior view for the nasal width.

Bivariate correlation analysis of pairs of bony and facial soft tissue sections in male and female groups revealed 45 pairs for males and 38 for females with Pearson’s correlation coefficients > |0.4|. Regression equations were developed from the measurements (Supplementary Table C). Using the regression equations, sections considered most effective for craniofacial reconstruction were identified (Tables 1 and 2). Except for the nasal width sections (bony sections N30 and N54, with their paired facial sections), the coefficients for the regression equations were relatively high. Therefore, these regression equations should be useful for prediction of nose morphology.

**Table 1:** Regression equations developed from measurements in males.

Midline		Bilateral	
Regression equation	R^2^ (%)	Regression equation	R^2^ (%)
N17 = 0.92 × N4 − 3.58	36	N45 = 0.66 × N35 − 3.97	61
N18 = 0.91 × N5 − 6.84	70	N69 = 0.62 × N59 − 2.63	52
N19 = 0.91 × N5 + 5.81	81	N46 = 0.75 × N35 + 3.07	54
N20 = 0.93 × N6 + 11.28	90	N70 = 0.75 × N59 + 3.70	51
N21 = 0.96 × N7 + 24.70	89	N47 = 0.66 × N35 + 6.72	60
N22 = 0.96 × N7 + 11.20	89	N71 = 0.66 × N59 + 6.77	59
		N48 = 0.66 × N35 + 14.01	65
		N72 = 0.69 × N59 + 13.52	67
		N49 = 0.91 × N39 + 19.98	85
		N73 = 0.92 × N63 + 18.62	84
		N50 = 0.95 × N39 + 10.59	90
		N74 = 0.99 × N63 + 8.11	90
		N51 = 0.98 × N39 + 12.26	87
		N75 = 1.02 × N63 + 9.19	88
		N52 = 0.96 × N39 + 15.18	88
		N76 = 0.99 × N63 + 13.23	88

The coefficient of determination is R^2^. All regression equations are shown in Supplementary Table C. Midline regression equations are paired with (a) and (b) in Fig. 3. Bilateral regression equations are paired with (g) to (i) in Fig. 3

**Table 2:** Regression equations developed from measurements in females.

Midline		Bilateral	
Regression equation	R^2^ (%)	Regression equation	R^2^ (%)
N17 = 0.85 × N4 − 1.10	35	N45 = 0.67 × N35 − 3.71	68
N18 = 1.01 × N5 − 9.04	68	N69 = 0.66 × N59 − 3.60	61
N19 = 1.00 × N5 + 3.23	85	N46 = 0.80 × N35 + 3.27	57
N20 = 0.96 × N6 + 8.36	96	N70 = 0.77 × N59 + 3.85	67
N21 = 1.00 × N7 + 19.50	93	N47 = 0.65 × N35 + 6.73	58
N22 = 1.02 × N7 + 5.18	92	N71 = 0.78 × N59 + 3.82	78
		N48 = 0.65 × N35 + 13.58	69
		N72 = 0.65 × N59 + 13.15	74
		N49 = 0.97 × N39 + 14.07	93
		N73 = 0.90 × N63 + 18.81	95
		N50 = 0.95 × N39 + 10.26	97
		N74 = 1.00 × N63 + 6.55	96
		N51 = 0.95 × N39 + 12.71	95
		N75 = 1.03 × N63 + 7.54	94
		N52 = 0.99 × N39 + 11.45	99
		N76 = 1.02 × N63 + 9.06	95

The coefficient of determination is R^2^. All regression equations are shown in Supplementary Table C. Midline regression equations are paired with (a) and (b) in Fig. 3. Bilateral regression equations are paired with (g) to (i) in Fig. 3

Supplementary Table B summarises the descriptive analysis of the 76 measurements, including means, standard variations, maximum values, and minimum values of each measurement.

### Landmark position comparison

Alar curvature superior, alar curvature inferior and alar curvature posterior are the landmarks lying on the groove of ala nasi. The positions of these landmarks have constant relationship with other landmarks on the nasal aperture groove.

**Figure 4:**
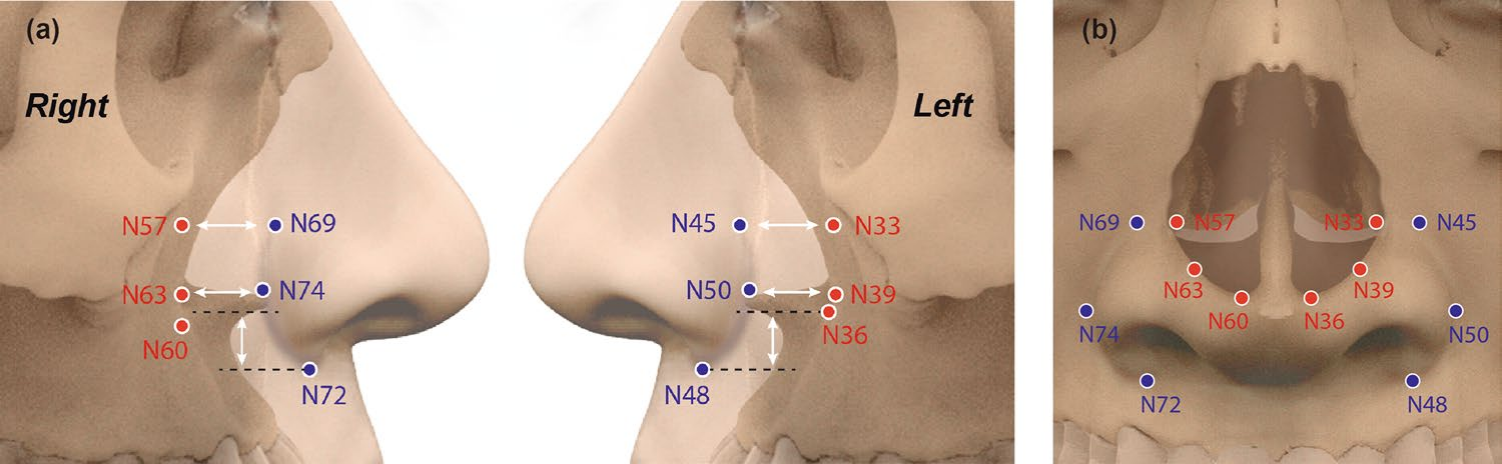
Comparison of positions of facial landmarks and adjacent bony landmarks. Anterior (**a**) and profile (**b**) views of alar curvature superior (N45, N69), inferior concha (N33, N57), alar curvature posterior (N50, N74), nasal aperture groove (N39, N63), alar curvature inferior (N48, N72), and nasal aperture inferior (N36, N60).

The left and right alar curvature superiors (N45, N69) lie about 1.4 mm inferior to the inferior conchae (N57, N33). The left and right alar curvature inferiors (N48, N72) lie about 3.2 mm inferior to the nasal aperture inferiors (N36, N60). The left and right alar curvature posteriors (N50, N74) lie approximately 6.9 mm anterior to the nasal aperture grooves (N63, N39). (Fig. 4, Table 3).

**Table 3:** Descriptive statistics of the landmark in nasal groove and their adjacent landmarks in nasal aperture.

A				B				|A − B|
Landmark	Position	Mean (mm)	SD	Landmark	Position	Mean (mm)	SD	
Alar curvature superior	N45 (left)	10.8	3.1	Inferior concha	N33 (left)	12.3	2.6	1.5
	N69 (right)	10.8	3.2		N57 (right)	12.1	2.8	1.3
Alar curvature inferior	N48 (left)	28.5	3.1	Nasal aperture inferior	N36 (left)	25.5	2.8	3.3
	N72 (right)	28.3	3.2		N60 (right)	25.2	2.9	3.1
Alar curvature posterior	N50 (left)	79.8	6.5	Nasal aperture groove	N39 (left)	73.0	6.4	6.8
	N74 (right)	80.1	6.7		N63 (right)	73.1	6.5	7.0

|A − B| Absolute value of the landmark position differences.

### Maximum width ratio

The maximum nasal width (MNW) was positively correlated with the maximum aperture width (MAW) (R = 0.35) at the 0.05 significance level; the ratios of MNW/MAW were 1.65 overall, 1.67 in males, and 1.58 in females. The ratios were similar to reported results^7,12^ for non-Koreans, except in females, although our female group was small (n = 22) and the correlation was weak (R = 0.19) (Table 4).

**Table 4:** Mean maximum nasal width (MNW), mean maximum aperture width (MAW), and the ratio of MNW/MAW in three groups (overall, in males, and in females).

	Mean MNW (mm)	Mean MAW (mm)	MNW/MAW
Overall	40.37	24.55	1.64
Males	41.15	24.72	1.66
Females	37.62	23.97	1.57

## Discussion

This study measured the nose and nasal aperture features of 100 Koreans to obtain detailed data for craniofacial reconstruction of the Korean population. Many distance measurements of the nose were strongly correlated with the morphologies of the skull and nasal aperture.

Unlike traditional research regarding nose morphology^6,12^, this study examined a broader range of faces and skulls. Rynn et al. and Stephan et al. generated regression equations for prediction of nose projection from nose and nasal aperture measurements of the faces and skulls^6,12^, while we measured distances between the landmarks and reference planes in a wide spectrum of faces and skulls, as shown in Fig. 2. The coronal reference plane passes through bregma, which is located where the coronal and sagittal sutures meet; this plane divides the head and skull into anterior and posterior sections. The orbital reference plane passes through the orbitale, located in the orbit; this plane separates the head and skull into superior and inferior sections. The distances from these planes to the landmarks were sufficiently long and included considerable portions of the face and skull.

The height of the nose to the orbital plane was correlated with the heights of the nasal aperture and nasal bone; the positions of the acanthion and nasion from the orbital plane were correlated with the positions of the pronasale, subnasale, and selion to yield the height of the nose. The depth of the nose from the coronal plane was correlated with the depths of the nasal aperture and nasal bone; the positions of the acanthion and nasion, relative to the coronal plane, were correlated with the positions of the pronasale, subnasale, and selion to yield the depth of the nose. The heights of the left and right ala nasi from the orbital reference plane in the anterior view were correlated with the nasal aperture groove. The distances from the left and right ala nasi to the midsagittal reference plane in the anterior view were correlated with the posterior alar curvature.

In the width of the nose and nasal aperture features, only the distance of the left and right alare and the left and right alar curvature posterior to the midsagittal plane were correlated. In other estimation, the correlation coefficient was high (Supplementary Table A), indicating low variability and a high probability of the correlation of the nose tissue and nasal aperture morphology.

The soft tissues of the human body undergo many changes immediately after death. Before post-mortem decomposition occurs, most of the changes in humans are related to skin colour (e.g., pallor mortis and livor mortis), due to changes in the circulation and actions of enzymes in muscle tissues, rather than changes in their volumes. In the rigor mortis stage, volumetric changes in humans result from muscle shortening. However, the reported variance is not statistically significant^23,24^. The nose has very small nasal muscles that result in a small volumetric change in the nasal region, compared with other parts of the human body.

Gravity can affect the ala nasi and nasal region if the subject undergoes CT in supine position, causing deformation of the nasal soft tissues. Bulut et al. investigated how the facial soft tissue thickness is influenced by the position of the head (i.e., upright versus supine)^25^. They used 3D laser scanned facial images to compare two positions, which revealed that the largest soft tissue deviations occurred in the mouth, cheeks, and masseter region because of downward sagging of the flesh caused by gravity. However, deviation near the nose was not statistically significant. Moreover, Ozsoy et al. examined 3D positional differences of the facial soft tissues; they found that the midsagittal landmarks (e.g., nasion, pronasale, and subnasale) were more stable than bilateral landmarks in the sitting, standing, and supine positions^26^. The nasal region is affected less by the subject’s position and the error is not statistically significant. Therefore, our results regarding the nose are reliable, although future studies should verify the results in living subjects.

While traditional methods focused on the projection of the nose^7,8,11^, our method also predicts the form and position of the nose and ala nasi in the anterior and lateral views. In profile, the projection of nose and depth of the left and right ala nasi are predictable. In the anterior view, the heights of the nose and left and right ala nasi are also predictable.

The regression equations can reliably predict the pronasale, subnasale, and selion for the nasal projection, yielding the form and position of the lateral nose. However, this method is useful for predicting the pronasale as a point on the nose surface, similar to another regression equation method^11^; the remaining nasal tip cannot be predicted from this method and errors might occur during prediction of the nasal tip area. Combining this with the two-tangent rule of Gerasimov, as described by Rynn et al.^12^, could correct for errors that result from regression equation methods to minimise errors in the prediction of nose morphology.

The rhinion, acanthion, and nasal aperture groove were each correlated with the ala nasi position in profile, using the coronal reference plane. The nasal aperture groove had the strongest correlation. When the nasal aperture groove is in poor condition, its regression equation can be replaced by the equations of the rhinion or acanthion for predicting the ala nasi (Fig. 3).

Rynn et al. found a correlation between the alar groove and nasal aperture features^12^. We used the same landmarks to define the relationship in Koreans. The alar curvature superiors are 1.5 mm left and 1.3 mm right inferior to the inferior nasal conchae, which are similar to the 1.1 mm differences in these two landmarks described by Rynn et al.^12^. The alar curvature inferiors are 3.3 mm left and 3.1 mm right inferior to the inferior nasal aperture, which are also similar to the 3.8-mm differences in these landmarks described by Rynn et al. The posterior alar curvatures are 6.8 mm left and 7.0 mm right anterior to the nasal aperture groove; these are similar to the 6.5 mm difference described by Rynn et al. (Fig. 4, Table 3). Therefore, the alar groove can be predicted from the nasal aperture pattern.

Rynn et al. included an Asian subgroup in their North American subjects^12^; however, their ethnic origins were unclear, and they might have differed from our Korean subjects. Despite this difference in origins, the relationships of each pair of landmarks to the alar groove and nasal aperture were similar to those in Rynn et al.^12^. In the vertical direction, the alar curvature superior and inferior nasal concha are nearly the same distance from the orbital plane; the alar curvature inferior is approximately 3 mm inferior to the inferior nasal aperture to the orbital plane. In the horizontal direction, the alar curvature posterior is approximately 6.9 mm anterior to the nasal aperture groove to the coronal plane. These findings indicate that the ala nasi phenotype matches the pattern of the nasal aperture, independent of ethnicity.

The maximum nasal width (MNW) was weakly (R = 0.35) correlated with the maximum aperture width (MAW). Rynn et al. reported a correlation coefficient of R = 0.58 and a ratio of MAW × 1.65 = MNW, which was comparable to the findings described by Gerasimov (MAW × 1.67 = MNW). Rynn et al. designated the estimation of MNW from the aperture morphology Gerasimov’s “Three-fifth rule” (i.e., MNW is estimated by multiplication of MAW by 5/3 or 1.67^12^. We obtained a similar MNW/MAW ratio (Table 4). The ratio value slightly varied among subgroups (European, Central Asian, or African ancestry) in the study by Rynn et al., but the overall ratio was 1.65 at the 0.05 significance level. The applicability of the “Five-thirds rule” to the Korean population is unclear, given the weak correlation and difference of the ratio between sexes.

The set of regression equations obtained from anthropometric analysis can be used to predict nose morphology. Forensic science uses various anthropometric analyses to predict nose morphology for craniofacial reconstruction^6,13–15^. Our results can be applied to Koreans and compared with other ethnic groups.

In plastic and reconstructive surgery, complex nasal reconstruction often requires symmetry and referential volume^27^. The application of nasal anthropometric analysis is very useful in such instances. Ballin et al. discussed the use of anthropometric measurements and calculation of the ratio to evaluate facial asymmetry before rhinoplasty^28^. This helps surgeons to assess the presence of nasal and facial asymmetry at the time of surgery^28^. Objective nose anthropometric measurements that yield the shape and size of the external nose help to determine surgical outcomes in paediatric reconstructive septoplasty and rhinoplasty for cleft lip nasal deformity; they also aid in the evaluation and characterisation of dysmorphic syndromes^29^.

This analysis can be applied to examine ethnic characteristics and differences in nasal form. Liu et al. reported differences in the morphology of the nose and other facial components between Chinese and African Americans during measurement of anthropometric landmarks^30^. Farkas et al. established facial databases for various ethnic groups^31^. Our data are valuable for understanding Korean nasal morphology, in the context of existing research findings.

## Conclusions

The nose on the face and nasal aperture of the skull are significantly correlated in terms of the overall face and skull morphology in Korean subjects. The ala nasi phenotype matches the pattern of the nasal aperture, independent of ethnicity. This finding differs from the results of previous studies regarding the relationships of nose and nasal aperture morphologies in a broader range of faces and skulls. Using these correlations, specific regression equations for Koreans were developed; these equations are expected to be useful for craniofacial reconstruction, with better reliability than other methods. The correlations between the morphology of the nasal aperture and nose can be used in clinical applications involving rhinoplasty and nasal reconstructive surgery. In a bio-anthropological context, the results might improve our understanding of nasal formation in relation to the overall structure of the skull in human evolution and associated ethnic variations.

## Methods

### Ethics approval

All methods performed in this study complied with the Declaration of Helsinki and were approved by the Institutional Review Board (IRB) of the National Forensic Service (IRB approval number: 906-180118-h-007-02).

### Subject selection and post-mortem computed tomography

The craniofacial samples studied were from 100 Koreans autopsied at the National Forensic Service Seoul Institute (NFS Seoul Institute) between March 2017 and September 2018.

The subjects comprised 78 male and 22 female Korean nationals. They ranged in age between 19 and 49 (mean 34.84) years to minimise the influence of changes in nose morphology due to aging. The subjects were divided into six groups according to sex and age. All subjects arrived at NFS Seoul Institute within 48 h of death. Subjects with marked changes in the morphology of the head or face due to illness or the cause of death were excluded, as were individuals with congenital malformations or prosthetics in their nose and nasal aperture areas.

The subjects were scanned using a SOMATOM Definition AS + (Siemens Healthineers, Erlangen, Germany). Because all autopsy procedures at NFS Seoul Institute are performed under a court-approved warrant, the need for informed consent from the next of kin was waived. For the CT images selected for this study, the head portions were segmented and exported to a research computer after all biological and personal information had been removed, except for the age and sex of the subjects. When measurement data had been collected, the exported head CT dataset was deleted.

### Craniofacial measurements

Mimics ver. 15.0 (Materialise, Leuven, Belgium) was used to apply the landmarks and reference planes and measure the distances. In total, 24 craniofacial landmarks [13 craniometric (bony) and 11 cephalometric (facial)] and seven reference planes were used to examine the morphometry of the nose and nasal aperture in 3D space (Fig. 1). The landmarks and reference planes are defined in Supplementary Table D. The position value of each landmark was the shortest distance from each reference plane (i.e., the perpendicular distance). Seventy-six distances were measured between landmarks and reference planes (Fig. 2; Supplementary Table E).

### Statistical analysis

Data were analysed using SPSS ver. 17.0 (SPSS, Chicago, IL, USA). Descriptive analysis was performed and the correlations of each measurement were calculated. *P* values < 0.05 were considered statistically significant.

## Supplementary information

Supplementary Tables.
