# Supplementary material for: Craniofacial anthropometric investigation of relationships between the nose and nasal aperture using 3D computed tomography of Korean subjects
Source: Sci Rep. 2020 Sep 30;10:16077. doi: 10.1038/s41598-020-73127-8 (PMC7527952; doi:10.1038/s41598-020-73127-8)
Supplement: Supplementary file 1 — Supplementary Tables. [file 41598_2020_73127_MOESM1_ESM.pdf]

Craniofacial anthropometric investigation of relationships between the nose and nasal aperture using 3D computed tomography of Korean subjects.

Joon Yeol Ryu[1], Ki-Su Park[1], Min-Ji Kim[1], Ji-Su Yun[1], U-Young Lee[2], Sang-Seob Lee[1], Byung-Yoon Roh[1], Jeong-Uk Seo[1], Chang-Un Choi[3], Won-Joon Lee[3*]

[1]Medical Examiner's Office, National Forensic Service, Wonju, 26460, Republic of Korea
[2]Catholic Institute for Applied Anatomy, Department of Anatomy, College of Medicine, Catholic University of Korea, Seoul, 06591, Republic of Korea
[3]Department of Forensic Medicine Investigation, National Forensic Service Seoul Institute, Seoul, 08063, Republic of Korea
*Corresponding author: ttolong2@gmail.com

Supplementary Table A

Correlations of the landmark position values

Direction	Section 1	Section 2	Relationship	Correlation coefficient (r)
Nasal height	N4	N17	Positive correlation	0.610
	N5	N18	Positive correlation	0.837
	N5	N19	Positive correlation	0.910
Nasal depth	N6	N20	Positive correlation	0.965
	N7	N21	Positive correlation	0.958
	N7	N22	Positive correlation	0.961
	N7	N49	Positive correlation	0.927
	N7	N50	Positive correlation	0.933
	N7	N51	Positive correlation	0.941
	N7	N52	Positive correlation	0.941
	N7	N73	Positive correlation	0.914
	N7	N74	Positive correlation	0.930
	N7	N75	Positive correlation	0.936
	N7	N76	Positive correlation	0.941
	N28	N49	Positive correlation	0.900
	N28	N50	Positive correlation	0.880
	N28	N51	Positive correlation	0.903
	N28	N52	Positive correlation	0.874
	N28	N73	Positive correlation	0.893
	N28	N74	Positive correlation	0.881
	N28	N75	Positive correlation	0.898
	N28	N76	Positive correlation	0.871
	N39	N49	Positive correlation	0.946
	N39	N50	Positive correlation	0.966
	N39	N51	Positive correlation	0.953
	N39	N52	Positive correlation	0.957
	N63	N73	Positive correlation	0.946
	N63	N74	Positive correlation	0.966
	N63	N75	Positive correlation	0.958
	N63	N76	Positive correlation	0.958
Ala nasi height	N35	N45	Positive correlation	0.791
	N35	N46	Positive correlation	0.727
	N35	N47	Positive correlation	0.777
	N35	N48	Positive correlation	0.817
	N59	N69	Positive correlation	0.739
	N59	N70	Positive correlation	0.734
	N59	N71	Positive correlation	0.799
	N59	N72	Positive correlation	0.826
Nasal width	N30	N42	Positive correlation	0.434
	N54	N66	Positive correlation	0.412

Supplementary Table B

Descriptive statistics analysis

Measurement code		Number	Minimum value (mm)	Maximum value (mm)	Mean (mm)	Standard deviation
**Nose**	**1**	100	-3.96	2.83	0.2504	1.27900
**Nose**	**2**	100	8.80	16.81	12.4007	1.55826
**Nose**	**3**	100	8.65	16.36	12.1536	1.53145
**Nose**	**4**	100	23.11	34.22	29.1426	2.50002
**Nose**	**5**	100	15.74	33.80	25.7314	3.26104
**Nose**	**6**	100	55.30	86.65	75.9797	6.18992
**Nose**	**7**	100	60.81	94.89	82.3236	6.83819
**Nose**	**8**	100	-2.50	2.33	-0.0337	0.85402
**Nose**	**9**	100	9.04	15.47	12.1167	1.38998
**Nose**	**10**	100	9.18	16.66	12.4375	1.18597
**Nose**	**11**	100	-5.06	3.46	-0.2510	1.71576
**Nose**	**12**	100	7.00	16.41	11.8995	1.92173
**Nose**	**13**	100	8.91	17.23	12.6550	1.87290
**Nose**	**14**	100	-2.86	2.83	-0.0651	1.19852
**Nose**	**15**	100	8.46	16.38	12.0854	1.60764
**Nose**	**16**	100	9.43	15.47	12.4686	1.34222
**Nose**	**17**	100	13.82	30.92	23.2658	3.52114
**Nose**	**18**	100	6.78	26.31	16.5701	3.58648
**Nose**	**19**	100	17.92	36.57	29.1458	3.36161
**Nose**	**20**	100	62.61	91.76	81.7034	6.22811
**Nose**	**21**	100	78.22	116.09	102.8662	7.35223
**Nose**	**22**	100	64.48	103.90	90.0269	7.24010
**Nose**	**23**	100	0.23	15.98	5.0065	3.11291
**Nose**	**24**	100	0.20	13.42	4.6473	2.95808
**Nose**	**25**	100	11.53	25.80	18.4794	2.85301
**Nose**	**26**	100	23.55	35.16	30.5166	2.71979
**Nose**	**27**	100	27.03	40.03	33.9316	2.79521
**Nose**	**28**	100	67.20	98.61	87.7128	6.52239
**Nose**	**29**	100	9.05	14.55	11.5093	1.06916
**Nose**	**30**	100	10.07	15.16	12.1504	1.14035
**Nose**	**31**	100	6.98	14.42	11.4468	1.73538
**Nose**	**32**	100	3.11	8.55	5.8688	1.18351
**Nose**	**33**	100	4.99	16.94	12.2503	2.56860
**Nose**	**34**	100	4.04	22.05	15.7166	3.16340
**Nose**	**35**	100	12.19	29.15	22.1927	3.74516
**Nose**	**36**	100	16.75	32.40	25.4589	2.80462
**Nose**	**37**	100	55.10	84.84	73.5619	6.27404
**Nose**	**38**	100	54.24	86.31	73.3330	6.44051
**Nose**	**39**	100	52.83	84.95	72.9743	6.43436
**Nose**	**40**	100	51.95	86.36	73.5235	6.57743
**Nose**	**41**	100	8.68	20.21	14.1987	2.34919

**Nose**	**42**	100	16.56	24.60	20.4200	1.77872
**Nose**	**43**	100	15.93	23.90	20.1708	1.80159
**Nose**	**44**	100	9.39	18.71	14.1622	1.97824
**Nose**	**45**	100	2.59	18.28	10.7627	3.08518
**Nose**	**46**	100	7.71	29.95	19.9580	3.78843
**Nose**	**47**	100	11.26	28.67	21.2487	3.16376
**Nose**	**48**	100	18.34	35.00	28.5278	3.08510
**Nose**	**49**	100	64.03	96.39	85.6815	6.54173
**Nose**	**50**	100	60.00	91.79	79.7878	6.45193
**Nose**	**51**	100	63.42	97.39	83.6086	6.92030
**Nose**	**52**	100	61.67	97.15	84.9495	6.87152
**Nose**	**53**	100	8.17	14.44	11.7332	1.04629
**Nose**	**54**	100	9.60	14.47	12.4037	0.99791
**Nose**	**55**	100	8.02	15.19	11.8260	1.64486
**Nose**	**56**	100	4.29	8.68	6.3312	1.08990
**Nose**	**57**	100	4.92	18.29	12.1096	2.78052
**Nose**	**58**	100	6.30	22.62	15.7906	3.02983
**Nose**	**59**	100	10.53	30.68	21.8411	3.81713
**Nose**	**60**	100	16.99	31.84	25.2156	2.89928
**Nose**	**61**	100	55.95	85.44	73.6820	6.38831
**Nose**	**62**	100	53.89	85.94	73.2899	6.49733
**Nose**	**63**	100	53.19	84.79	73.1534	6.52684
**Nose**	**64**	100	51.86	84.83	73.6811	6.59156
**Nose**	**65**	100	10.38	21.73	14.5044	2.21573
**Nose**	**66**	100	16.40	26.12	20.5677	1.85323
**Nose**	**67**	100	16.52	25.10	20.2031	1.84877
**Nose**	**68**	100	9.91	18.79	14.4062	1.89636
**Nose**	**69**	100	2.83	18.37	10.7828	3.23381
**Nose**	**70**	100	7.70	29.31	20.2672	3.90412
**Nose**	**71**	100	11.91	28.03	21.0408	3.30398
**Nose**	**72**	100	18.21	34.63	28.2592	3.19682
**Nose**	**73**	100	65.86	97.69	85.6194	6.48986
**Nose**	**74**	100	58.70	92.83	80.0919	6.72722
**Nose**	**75**	100	60.65	97.33	83.4889	7.15313
**Nose**	**76**	100	59.78	99.41	84.9098	7.07190

Supplementary Table C

Regression equations developed from the measurements in male group.

Midline			
**Dependent Variable**	**Independent Variable**	**Regression equation**	R^2
**N4**	**N17**	**N17**=0.92***N4**-3.58	36%
**N5**	**N18**	**N18**=0.91***N5**-6.84	70%
**N5**	**N19**	**N19**=0.91***N5**+5.81	81%
**N6**	**N20**	**N20**=0.93***N6**+11.28	90%
**N7**	**N21**	**N21**=0.96***N7**+24.70	89%
**N7**	**N22**	**N22**=0.96***N7**+11.20	89%
**Bilateral**			
**Dependent Variable**	**Independent Variable**	**Regression equation**	R^2
**N7**	**N49**	**N49**=0.84***N7**+16.41	80%
	**N73**	**N73**=0.83***N7**+17.20	75%
**N7**	**N50**	**N50**=0.87***N7**+8.61	82%
	**N74**	**N74**=0.90***N7**+6.34	81%
**N7**	**N51**	**N51**=0.92***N7**+8.38	82%
	**N75**	**N75**=0.94***N7**+6.12	81%
**N7**	**N52**	**N52**=0.90***N7**+11.41	83%
	**N76**	**N76**=0.92***N7**+9.52	83%
**N28**	**N49**	**N49**=0.85***N28**+11.47	71%
	**N73**	**N73**=0.85***N28**+10.92	69%
**N28**	**N50**	**N50**=0.83* **N28**+7.11	66%
	**N74**	**N74**=0.87***N28**+4.21	66%
**N28**	**N51**	**N51**=0.90* **N28**+4.58	70%
	**N75**	**N75**=0.94***N28**+1.61	70%
**N28**	**N52**	**N52**=0.83***N28**+12.30	63%
	**N76**	**N76**=0.85***N28**+10.92	62%
**N35**	**N45**	**N45**=0.66***N35**-3.97	61%
**N59**	**N69**	**N69**=0.62***N59**-2.63	52%
**N35**	**N46**	**N46**=0.75***N35**+3.07	54%
**N59**	**N70**	**N70**=0.75***N59**+3.70	51%
**N35**	**N47**	**N47**=0.66***N35**+6.72	60%
**N59**	**N71**	**N71**=0.66***N59**+6.77	59%
**N35**	**N48**	**N48**=0.66***N35**+14.01	65%
**N59**	**N72**	**N72**=0.69***N59**+13.52	67%
**N39**	**N49**	**N49**=0.91***N39**+19.98	85%
**N63**	**N73**	**N73**=0.92***N63**+18.62	84%
**N39**	**N50**	**N50**=0.95***N39**+10.59	90%
**N63**	**N74**	**N74**=0.99***N63**+8.11	90%
**N39**	**N51**	**N51**=0.98***N39**+12.26	87%
**N63**	**N75**	**N75**=1.02***N63**+9.19	88%
**N39**	**N52**	**N52**=0.96***N39**+15.18	88%
**N63**	**N76**	**N76**=0.99***N63**+13.23	88%
**N30**	**N41**	**N41**=0.56***N30**+7.61	7%
**N54**	**N65**	($p \geq 0.05$)	
**N30**	**N42**	**N42**=0.68***N30**+12.51	19%
**N54**	**N66**	**N66**=0.79***N54**+11.13	17%

N30	N43	N43=0.58*N30+13.39	15%
N54	N67	N67=0.68*N54+12.12	13%
N30	N44	N44=0.65*N30+6.57	14%
N54	N68	N68=0.48*N54+8.83	7%

Variable 1 indicates the measurement sections in the skull and variable 2 indicates the measurement sections in the facial soft tissue.

Regression equations developed from the measurements in female group.

Midline			
**Dependent Variable**	**Independent Variable**	**Regression equation**	**R^2**
N4	N17	N17=0.85*N4-1.10	35%
N5	N18	N18=1.01*N5-9.04	68%
N5	N19	N19=1.00*N5+3.23	85%
N6	N20	N20=0.96*N6+8.36	96%
N7	N21	N21=1.00*N7+19.50	93%
N7	N22	N22=1.02*N7+5.18	92%
**Bilateral**			
**Dependent Variable**	**Independent Variable**	**Regression equation**	**R^2**
N7	N49	N49=0.95*N7+7.65	86%
	N73	N73=0.93*N7+9.62	91%
N7	N50	N50=0.93*N7+3.83	90%
	N74	N74=1.03*N7-3.54	91%
N7	N51	N51=0.95*N7+4.62	93%
	N75	N75=1.07*N7-3.82	92%
N7	N52	N52=0.97*N7+4.68	89%
	N76	N76=1.05*N7-1.35	91%
N28	N49	N49=0.96*N28+1.71	87%
	N73	N73=0.92*N28+4.71	89%
N28	N50	N50=0.92*N28-0.61	87%
	N74	N74=1.02*N28-8.55	89%
N28	N51	N51=0.96*N28-0.93	93%
	N75	N75=1.05*N28-8.27	88%
N28	N52	N52=0.96*N28+0.16	86%
	N76	N76=1.03*N28-5.60	87%
N35	N45	N45=0.67*N35-3.71	68%
N59	N69	N69=0.66*N59-3.60	61%
N35	N46	N46=0.80*N35+3.27	57%
N59	N70	N70=0.77*N59+3.85	67%
N35	N47	N47=0.65*N35+6.73	58%
N59	N71	N71=0.78*N59+3.82	78%
N35	N48	N48=0.65*N35+13.58	69%
N59	N72	N72=0.65*N59+13.15	74%
N39	N49	N49=0.97*N39+14.07	93%
N63	N73	N73=0.90*N63+18.81	95%
N39	N50	N50=0.95*N39+10.26	97%
N63	N74	N74=1.00*N63+6.55	96%
N39	N51	N51=0.95*N39+12.71	95%
N63	N75	N75=1.03*N63+7.54	94%
N39	N52	N52=0.99*N39+11.45	99%

**N63**	**N76**	**N76**=1.02***N63**+9.06	95%
**N30**	**N41**	($p \geq 0.05$)	
**N54**	**N65**		
**N30**	**N42**		
**N54**	**N66**		
**N30**	**N43**	($p \geq 0.05$)	
**N54**	**N67**		
**N30**	**N44**		
**N54**	**N68**		

Variable 1 indicates the measurement sections in the skull and variable 2 indicates the measurement sections in the facial soft tissue.

Supplementary Table D

Definition of landmarks

Abbreviation	Landmark name	Definition
	**Median landmark**	
**N**	Nasion	Intersection of the nasofrontal sutures in the median plane
**R**	Rhinion	Most rostral (end) point on the internasal suture. Cannot be determined accurately if nasal bones are broken distally
**AC**	Acanthion	Most anterior tip of the anterior nasal spine
**S**	Selion	Deepest midline point of the nasofronal angle
**PN**	Pronasale	Most anteriorly projected point on a nose
**SN**	Subnasale	Median point at the junction between the lower border of the nasal septum and the philtrum area
	**Bilateral landmark**	
**O**	Orbitale	Most inferior point on the inferior orbital rim. Usually falls along the lateral half of the orbital margin
**IC**	Inferior nasal concha	Point where the inferior nasal concha is submerged into the medial wall of the nasal aperture
**A**	Alare	Instrumentally determined as the most lateral point on the nasal aperture in a transverse plane
**NAG**	Nasal aperture groove	Most posterior point on the lateral curvature of the nasal aperture from profile view
**NAI**	Nasal aperture inferior	Most inferior point on the nasal aperture from the frontal view
**ACS**	Alar curvature superior	Highest point on the left ala nasi(rounded wing shape of a nose)
**ACP**	Alar curvature posterior	Most posterolateral point of the curvature of the base of the nasal alae
**NA**	Nose alare	The most lateral point on the nasal ala
**ACI**	Alar curvature inferior	Most posterolateral point of the curvature of the base line of each nasal ala

Definition of reference plane

Reference plane	Definition
Midsagittal plane	Plane passing through the three landmarks, nasion, prosthion and lambda
Orbital plane	Plane that passes through the two landmarks auriculare midpoint and orbitale and is orthogonal to the midsagittal plane
Coronal plane	Plane that passes through one landmark, bregma and is orthogonal to the midsagittal plane and orbital plane
Rhinion plane	Plane that passes through one landmark, rhinion and is parallel to the orbital plane
Alare sagittal plane	Plane that passes through one landmark, alare and is parallel to the midsagittal plane

Supplementary Table E

Description of measurement sections in each measurement code

<table>
<tr><th colspan="2">Measurement code</th><th>Measurement section</th><th>Measurement location</th><th>Measurement direction</th></tr>
<tr><td>Nose</td><td>1</td><td>Acanthion - Midsagittal plane</td><td rowspan="5">Anteriority</td><td rowspan="3">Horizontality</td></tr>
<tr><td>Nose</td><td>2</td><td>Acanthion - Alare sagittal plane</td></tr>
<tr><td>Nose</td><td>3</td><td>Acanthion - Alare sagittal plane</td></tr>
<tr><td>Nose</td><td>4</td><td>Nasion - Orbital plane</td><td rowspan="2">Verticality</td></tr>
<tr><td>Nose</td><td>5</td><td>Acanthion - Orbital plane</td></tr>
<tr><td>Nose</td><td>6</td><td>Nasion - Coronal plane</td><td rowspan="2">Profile</td><td rowspan="11">Horizontality</td></tr>
<tr><td>Nose</td><td>7</td><td>Acanthion - Coronal plane</td></tr>
<tr><td>Nose</td><td>8</td><td>Selion - Midsagittal plane</td><td rowspan="12">Anteriority</td></tr>
<tr><td>Nose</td><td>9</td><td>Selion - Alare sagittal plane</td></tr>
<tr><td>Nose</td><td>10</td><td>Selion - Alare sagittal plane</td></tr>
<tr><td>Nose</td><td>11</td><td>Pronasale - Midsagittal plane</td></tr>
<tr><td>Nose</td><td>12</td><td>Pronasale - Alare sagittal plane</td></tr>
<tr><td>Nose</td><td>13</td><td>Pronasale - Alare sagittal plane</td></tr>
<tr><td>Nose</td><td>14</td><td>Subnasale - Midsagittal plane</td></tr>
<tr><td>Nose</td><td>15</td><td>Subnasale - Alare sagittal plane</td></tr>
<tr><td>Nose</td><td>16</td><td>Subnasale - Alare sagittal plane</td></tr>
<tr><td>Nose</td><td>17</td><td>Selion - Orbital plane</td><td rowspan="3">Verticality</td></tr>
<tr><td>Nose</td><td>18</td><td>Pronasale - Orbital plane</td></tr>
<tr><td>Nose</td><td>19</td><td>Subnasale - Orbital plane</td></tr>
<tr><td>Nose</td><td>20</td><td>Selion - Coronal plane</td><td rowspan="3">Profile</td><td rowspan="3">Horizontality</td></tr>
<tr><td>Nose</td><td>21</td><td>Pronasale - Coronal plane</td></tr>
<tr><td>Nose</td><td>22</td><td>Subnasale - Coronal plane</td></tr>
<tr><td>Nose</td><td>23</td><td>Orbitale - Rhinion plane</td><td rowspan="5">Anteriority</td><td rowspan="5">Verticality</td></tr>
<tr><td>Nose</td><td>24</td><td>Orbitale - Rhinion plane</td></tr>
<tr><td>Nose</td><td>25</td><td>Selion - Rhinion plane</td></tr>
<tr><td>Nose</td><td>26</td><td>Acanthion - Rhinion plane</td></tr>
<tr><td>Nose</td><td>27</td><td>Subnasale - Rhinion plane</td></tr>
<tr><td>Nose</td><td>28</td><td>Rhinion - Coronal plane</td><td>Profile</td><td rowspan="5">Horizontality</td></tr>
<tr><td>Nose</td><td>29</td><td>Inferior concha - Midsagittal plane</td><td rowspan="6">Anteriority</td></tr>
<tr><td>Nose</td><td>30</td><td>Alare - Median sagittal plane</td></tr>
<tr><td>Nose</td><td>31</td><td>Nasal aperture groove - Midsagittal plane</td></tr>
<tr><td>Nose</td><td>32</td><td>Nasal aperture inferior - Midsagittal plane</td></tr>
<tr><td>Nose</td><td>33</td><td>Inferior concha - Orbital plane</td><td rowspan="2">Verticality</td></tr>
<tr><td>Nose</td><td>34</td><td>Alare - Orbital plane</td></tr>
</table>

Nose	35	Nasal aperture groove - Orbital plane		
Nose	36	Nasal aperture inferior - Orbital plane		
Nose	37	Inferior concha - Coronal plane	Profile	Horizontality
Nose	38	Alare - Coronal plane		
Nose	39	Nasal aperture groove - Coronal plane		
Nose	40	Nasal aperture inferior - Coronal plane		
Nose	41	Alar curvature superior - Midsagittal plane	Anteriority	
Nose	42	Alar curvature posterior - Midsagittal plane		
Nose	43	Alare - Midsagittal plane		
Nose	44	Alar curvature inferior - Midsagittal plane		
Nose	45	Alar curvature superior - Orbital plane		Verticality
Nose	46	Alar curvature posterior - Orbital plane		
Nose	47	Alare - Orbital plane		
Nose	48	Alar curvature inferior - Orbital plane		
Nose	49	Alar curvature superior - Coronal plane	Profile	Horizontality
Nose	50	Alar curvature posterior - Coronal plane		
Nose	51	Alare - Coronal plane		
Nose	52	Alar curvature inferior - Coronal plane		
Nose	53	Inferior concha - Midsagittal plane	Anteriority	
Nose	54	Alare - Midsagittal plane		
Nose	55	Nasal aperture groove - Midsagittal plane		
Nose	56	Nasal aperture inferior - Midsagittal plane		
Nose	57	Inferior concha - Orbital plane		Verticality
Nose	58	Alare - Orbital plane		
Nose	59	Nasal aperture groove - Orbital plane		
Nose	60	Nasal aperture inferior - Orbital plane		
Nose	61	Inferior concha - Coronal plane	Profile	Horizontality
Nose	62	Alare - Coronal plane		
Nose	63	Nasal aperture groove - Coronal plane		
Nose	64	Nasal aperture inferior - Coronal plane		
Nose	65	Alar curvature superior - Midsagittal plane	Anteriority	
Nose	66	Alar curvature posterior - Midsagittal plane		
Nose	67	Alare - Midsagittal plane		
Nose	68	Alar curvature inferior - Midsagittal plane		
Nose	69	Alar curvature superior - Orbital plane		Verticality
Nose	70	Alar curvature posterior - Orbital plane		
Nose	71	Alare - Orbital plane		
Nose	72	Alar curvature inferior - Orbital plane		
Nose	73	Alar curvature superior - Coronal plane	Profile	Horizontality
Nose	74	Alar curvature posterior - Coronal plane		
Nose	75	Alare - Coronal plane		
Nose	76	Alar curvature inferior - Coronal plane		
